# Adaptation to life in aeolian sand: how the sandfish lizard, *Scincus scincus*, prevents sand particles from entering its lungs

**DOI:** 10.1242/jeb.138107

**Published:** 2016-11-15

**Authors:** Anna T. Stadler, Boštjan Vihar, Mathias Günther, Michaela Huemer, Martin Riedl, Stephanie Shamiyeh, Bernhard Mayrhofer, Wolfgang Böhme, Werner Baumgartner

**Affiliations:** 1 Institute of Biomedical Mechatronics, Johannes Kepler University Linz, Altenbergerstraße 69, Linz 4040, Austria; 2 Institute of Biology II, RWTH Aachen University, Worringerweg 3, Aachen 52074, Germany; 3 IRNAS, Drevesniška 25, Rače 2327, Slovenia; 4 Zoologisches Forschungsmuseum Alexander Koenig (ZFMK), Adenauerallee 160, Bonn 53 11 3, Germany

**Keywords:** *Scincus scincus*, Respiration, Calculation, Particle flow

## Abstract

The sandfish lizard, *Scincus scincus* (Squamata: Scincidae), spends nearly its whole life in aeolian sand and only comes to the surface for foraging, defecating and mating. It is not yet understood how the animal can respire without sand particles entering its respiratory organs when buried under thick layers of sand. In this work, we integrated biological studies, computational calculations and physical experiments to understand this phenomenon. We present a 3D model of the upper respiratory system based on a detailed histological analysis. A 3D-printed version of this model was used in combination with characteristic ventilation patterns for computational calculations and fluid mechanics experiments. By calculating the velocity field, we identified a sharp decrease in velocity in the anterior part of the nasal cavity where mucus and cilia are present. The experiments with the 3D-printed model validate the calculations: particles, if present, were found only in the same area as suggested by the calculations. We postulate that the sandfish has an aerodynamic filtering system; more specifically, that the characteristic morphology of the respiratory channel coupled with specific ventilation patterns prevent particles from entering the lungs.

## Abstract (precis)

**Highlighted Article:** The sandfish *S. scincus* spends nearly its whole life in fine desert sand. We discovered that it has an aerodynamic filtering system to prevent sand particles from entering the lungs.

## INTRODUCTION

The sandfish lizard, *Scincus scincus* (Linnaeus 1758), is a species of the family Scincidae living in the sandy deserts of North Africa and the Middle East (Hartmann, 1989). Its environment is characterised by extreme aridity, strong temperature oscillations and, in particular, high daytime temperatures. Morphological and physiological adaptations are essential for survival in such hostile surroundings.

To sustain physiological homeostasis, this poikilothermic animal adjusts its body temperature by moving between layers of loose sand, its main habitat; the skink only comes to the surface for foraging, defecating and mating. It moves through the sand in an almost fish-like manner, thus its common name is sandfish. The morphological adaptations of the locomotory apparatus and subsurface locomotion have been described and studied previously (Hartmann, 1989; Baumgartner et al., 2008; Maladen et al., 2009, 2011; Knight, 2012; Sharpe et al., 2013, 2015; Dorgan, 2015). In addition, it has been reported (Baumgartner et al., 2007; Staudt et al., 2011; Rechenberg et al., 2004) that the skin is highly resistant to abrasion and shows low friction with sand. The respiratory movements of fossorial lizards have been described by Pough (1969a,b, 1971). He observed that breathing is performed by vertical movements of the venter instead of lateral movement of the trunk, noticed in non-fossorial lizards. He concluded that the so-formed sand-free pocket under the venter makes breathing physically possible. We observed similar respiratory movements in *S. scincus* as Pough (1971) described for *Ophiomorus tridactylus*, another representative of the family Scincidae. Recently, the respiratory physiology of the sandfish and the physical properties of its natural habitat have been investigated (Vihar et al., 2015). Vihar et al. (2015) pointed out that, because of their irregular shapes, sand particles create interconnected interstitial pockets filled with sufficient air for the sandfish to remain below sand for prolonged periods.

The upper respiratory tract of *S. scincus* has not yet been described in detail. Mosauer (1932), who studied structure and behaviour of sand reptiles, was surprised not to find any special adaptation of the nostrils against sand in *S. scincus*. Stebbins (1943), inspired by Mosauer's observations, focused on the nasal structure of the sand-burrowing lizard *Uma notata*. He noticed that the animal inhaled sand particles but eventually exhaled them, and postulated that the characteristic U-shape of the nasal cavity prevents particles from entering the lungs. In conjunction with the previous study, Stebbins (1948) examined the nasal apparatus of several lizard families, among them one representative of the family Scincidae, genus *Eumeces*. He described the nasal organ of the family Scincidae as being poorly adapted to preventing the entrance of fine particles.

It is not yet understood how the sandfish can breathe without sand particles entering its lungs while buried in this granular medium.

## MATERIALS AND METHODS

### Animal models

All *S.* *scincus* specimens used in this work were wild-caught. Therefore, we could not determine the age of the animals. We did not determine the sex of the sandfish specimens, because this procedure is rather painful for them and we did not regard this parameter as relevant to our experiments.

According to the Veterinary Office, City of Bonn, Germany, the experiments we performed to study the ventilation patterns were considered as animal observations and hence not subject to authorisation.

List of symbols and abbreviations

a.c.: anterior chamber
*A_i_*: cross-sectional area (m^2^)
ao.s.: antiorbital space
b.b.: basal bodies
ch.d.: choanal duct
Co: concha
e.n.: external naris
e.t.: erectile tissue
*f*: magnification factor for helium atmosphere
***g***: gravitational acceleration (m s^–2^)
g.c.: goblet cells
*h*: height of the different sections of the upper respiratory tract (m)
i.n.: internal naris
*L*: characteristic length, in our case the diameter of the respiratory canal (m)
n.c.: nasal cavity
nph.: nasopharynx
o.c.: oral cavity
ol.c.: olfactory chamber
oph.: oropharynx
*p*: pressure in the nasal cavity (Pa)
p.: proprium
p.c.: palatine cleft
ph.: pharynx
r.c.: respiratory chamber
*Re*: Reynold's number
TEM: transmission electron microscopy
*u*: characteristic velocity (m s^–1^)
v.: valve
ve.: vestibulum
*v*_ie_: initial exhalation velocity (m s^–1^)
*v*_ii_: initial inhalation velocity (m s^–1^)
v-n.o.: vomero-nasal organ
*V*_T_: tidal volume (ml 100 g^–1^)
η: dynamic viscosity (Pa s)
ν: kinematic viscosity (m^2^ s^–1^)
ρ: density of the fluid (kg m^–3^)

### Dissection of sandfish specimens

In total, five specimens were dissected. Two of them were museum specimens; the other three were dissected after they had recently died. The specimens [from Zoologisches Forschungsmuseum Alexander Koenig (ZFMK), Germany] were opened ventrally, and the gastrointestinal tract was removed and studied for sand particles. The respiratory tract and both lung sacs were carefully opened with a scalpel, and the interior was examined for sand particles by means of a stereo microscope (Olympus Tokyo SZ, Tokyo, Japan). Furthermore, the total lung volume was determined (see Vihar et al. 2015). One sandfish head was embedded for histology.

### Histology of the upper respiratory tract

For histological examination, the head of one *S. scincus* specimen was embedded in epoxy resin: 13 g glycide ether, 5.5 g dodecenyl succinic anhydride (DDSA), 5.7 g methylnadic anhydride (MNA) and 0.15 g 2,4,6-Tris(dimethylaminomethyl)phenol (DMP) were slowly mixed with a magnetic stirrer until streaks disappeared (all chemicals from Serva, Heidelberg, Germany). The mixture was then degassed in a desiccator (Kartell, Milan, Italy).

The embedded tissue was cut into 5–10 μm semi-thin sections using a rotary microtome (Ultracut E, Reichert Jung, Wetzlar, Germany) with a d-profile knife (Jung, Wetzlar, Germany). Each slide was placed on a cover slip with a drop of double-distilled H_2_O in order to straighten the sample. To smooth and make the sample adhere to the glass while the water slowly evaporated, the cover slip was placed on a heating plate (150 W, Medax, Neumuenster, Germany) at 60°C for at least 8 h.

The method we chose for polychromatic staining is based on that by Tolivia et al. (1994) but without using osmium tetroxide to prepare the tissue. Solution A was prepared using 12.5 ml Carbol Gentian Violet (Waldeck, Muenster, Germany), 10 ml 96% Et-OH, 2.5 ml pyridine (both Roth, Karlsruhe, Germany), 12.5 ml distilled H_2_O and 12.5 ml Carbol Methylene Blue consisting of 2% Methylene Blue (Honeywell, Seelze, Germany) and 0.5% phenole (Merck, Darmstadt, Germany) dissolved in distilled H_2_O. Solution B was prepared by dissolving 5 g pararosaniline powder (Waldeck), 97 ml distilled H_2_O, 1 ml 100% acetic acid (Honeywell) and 2 ml 5% phenole (Merck). The samples were stained in solution A for 45 s and washed 45 s with distilled H_2_O. The samples were then stained for 45 s in solution B and washed with distilled H_2_O for 30 s. The stained samples were dried in air at room temperature for 5 days.

Before microscopic examination, the stained samples were embedded with a drop of Euparal (Waldeck). The samples were examined with a light microscope (BA300, Motic, Wetzlar, Germany) and digitised by means of a microscope camera (Moticam 2300, Motic).

To verify the presence of goblet cells, several samples were examined at higher magnification; cilia were observed on transmission electron microscopy (TEM) images we took from one of the sections.

Micro-computed tomography and magnetic resonance imaging were performed on two sandfish specimens; they confirmed the results of histology. For this and ethical reasons we chose not to histologically examine further *S. scincus* specimens.

### Analysis of flow dynamics

**Fig. 1.**
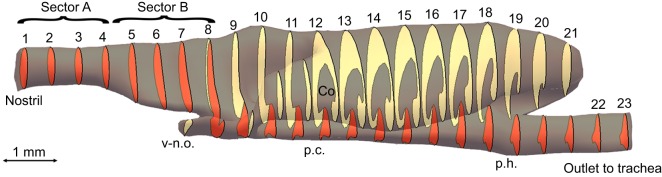
**Characteristic sections of the 3D model.** The model was divided into 23 evenly distributed sections to analyse the cross-sections and separate the olfactory and respiratory tracts according to the valve that is formed at the palatine cleft (p.c.). Co, concha; ph., pharynx; v-n.o., vomero-nasal organ.

**Fig. 2.**
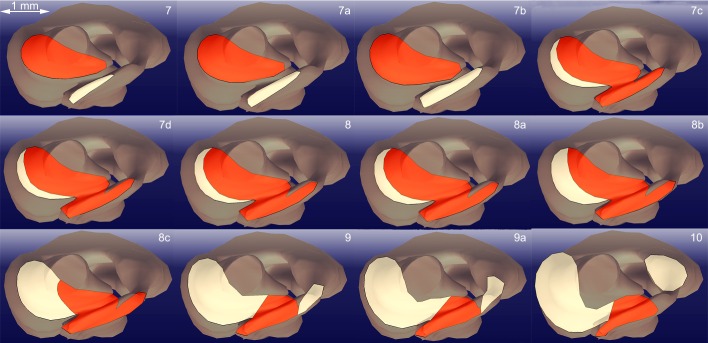
**Selected sections of the 3D model.** The respiratory and olfactory tracts are indicated in red and yellow, respectively. The sections show the sharp decrease in diameter of the respiratory tract posterior to the concha. In section 7d, the cross-sectional area of the respiratory tract is at its largest.

The digitised histological samples were merged using the 3D graphics software Blender (www.blender.org, OpenSource) to build a 3D model of one side of the bilateral structure of the entire upper respiratory system from nostrils to trachea. The model was then geometrically analysed with the CAD software CATIA (Computer Aided Three-dimensional Interactive Application, Version 5.17, Dassault Systémes, Vélizy-Villacoublay Cedex, France), and these data were implemented in MATLAB (R2014a, MathWorks, Natick, MA, USA), a multi-paradigm numerical computing environment, to calculate the mean flow velocity and pressure field (see Table S1). The model was divided into 31 sections, 23 of which were evenly distributed (Fig. 1), and the remaining eight were places in zones with substantial changes in cross-section (Fig. 2). The sectional areas of the respiratory channel were determined to calculate the mean velocity in each section.

The following considerations were taken into account in order to obtain an analytical solution for the velocity and pressure field. The canal was assumed to be a pipe, and the flow inside it was assumed to be steady and frictionless; the fluid was considered incompressible. These assumptions are justified if the flow is highly laminar.

To calculate the Reynolds number, the values for the density and dynamic viscosity of air were taken from the VDI-Waermeatlas (VDI e.V., 2013). The characteristic velocity was calculated from the tidal volume [determined by Vihar et al. (2015) to be 227±48 μl], the cross-sectional area, the point at which the flow streams in, and the average inhalation and exhalation times, respectively (see Eqn 7). The characteristic length was given by the diameter of each cross-section. The Reynolds numbers (see Eqn 3) for the very slow inhalation velocity and the rapid exhalation velocity were 10 and 562, respectively. Because the flow in a pipe is laminar for a Reynolds number below 2300, our assumptions were reasonable, and we were able to use the mass equation (Eqn 1) and the Bernoulli equation (Eqn 2) to determine the velocity and pressure field:
(1)
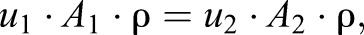

(2)
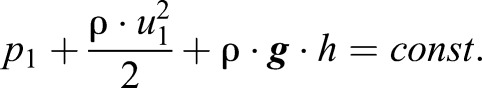



Here, *A* (m^2^) denotes the cross-sectional area of the corresponding section, *p* (Pa) the pressure, ***g*** (m s^–2^) the gravitational acceleration and *h* (m) the height.

To determine the height, the sections were divided into three parts. From sections 1 to 7b, *h* was set to zero, *h*=0 m; from sections 7c to 8c, the mean height was calculated to be *h*=–0.61×10^–3^ m, and from sections 9 to 23 the mean height was determined to be *h*=–0.98×10^–3^ m.

### Ventilation patterns

The respiration patterns of the lizard were recorded using a piezo sensor (Belusic and Zupancic, 2010). The piezo element was insulated with adhesive tape to prevent polarisation with sand, and connected with two copper cables. Connections were insulated with heat shrink tubes (all electronic equipment from Reichelt, Sande, Germany). A 3×3 mm^2^ piece of plastic was glued to the sensory membrane of the piezo element to concentrate the pressure created by touch. A piece of soft paper tissue prevented abrasion of the sandfish skin. The sensor was tightly attached to the skink's chest with adhesive tape forming a rigid loop around its thorax. For recording, the sensor was connected to a digital oscilloscope (PCSU1000, Velleman Instruments, Gavere, Belgium). The plethysmographic changes in the thoracic region were measured for 30 min with the animal at the surface and 30 min in terrarium sand [particle diameter: ∼0.1 to 0.5 mm in diameter, from Terra Exotica, Alfeld (Leine), Germany]. For both experiments, the sandfish specimen was kept in a terrarium (Terra Exotica) at room temperature (∼23°C), where it could move freely.

For comparison, we observed the ventilation patterns of *Eumeces schneideri* (Daudin 1802) (Squamata: Scincidae) at the surface; the results for *E. schneideri* and *S. scincus* were almost identical. *Eumeces* *schneideri* is a non-fossorial lizard, hence we could not observe the respiration patterns in terrarium sand.

### Fluid mechanics experiments

**Fig. 3.**
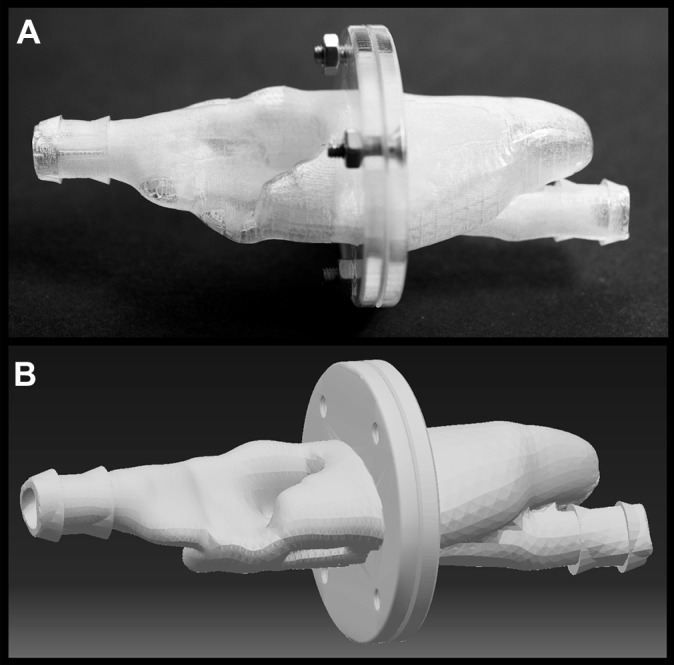
**The digital model and its realization.** (A) Printed model assembled with screws. The material is translucent, which enables visual examination for sand particles. (B) Digital model comprising two parts. The flange was designed for assembly after printing; tube adapters were added to the model to ensure a firm connection.

The model (one side of the symmetric upper respiratory tract) was printed with a 3D printer (Objet30 Pro, RTC Rapid Technologies, Mettmann, Germany) at a resolution of 600×600 dpi. The printing material (synthetic resin) was translucent. For handling purposes, the following modifications were introduced with CATIA: the model was separated into two parts, and a flange was added with four thread holes to allow the parts to be screwed together after printing. A tube adapter was added at the outlet to the trachea (Fig. 3). The original model was approximately 11 mm long and 3.5 mm wide at its widest point. Because of the complex and delicate structure of the respiratory system, we decided to scale up the model, which made printing and removing the support material after printing feasible and the model easier to handle. Further, this enabled visual examination of the object to search for sand particles after the experiment. Because the air flow was highly laminar, it was sufficiently accurate to design the experimental parameters considering the same Reynolds number as in the original size. This guaranteed the same or very similar flow behaviour. The Reynolds number (*Re*) is a dimensionless quantity and is defined as the ratio of momentum forces to viscous forces:
(3)
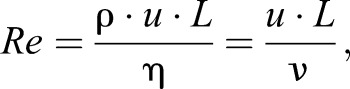

where ρ (kg m^–3^) is the density of the fluid; η (Pa s) is the dynamic viscosity; ν (m^2^ s^–1^) is the kinematic viscosity; *L* (m) denotes the characteristic length, in our case the diameter of the respiratory canal; and *u* (m s^–1^) is the characteristic velocity.

To scale up the model, the characteristic length *L* had to be changed by a factor *f* (Eqn 4). The characteristic velocity *u* was considered to be constant because it would have been difficult to modulate the respiration frequency. Consequently, we changed the gas atmosphere:
(4)
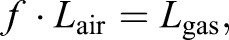

(5)
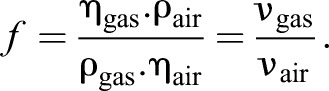



We calculated the most convenient magnification factor (*f*=7.8) for helium (at room temperature), where *f* denotes the ratio between the kinematic viscosities of helium and air:
(6)
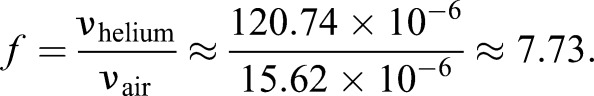



In Eqn 6 the factor *f* was calculated for 23°C. For the experiment we calculated the factor for 20, 23 and 25°C and determined the mean value to be *f*_mean_≈7.8. Values of dynamic viscosity and density of air were obtained from the VDI-Waermeatlas (VDI e.V., 2013), and for helium the values were acquired from the NIST Chemistry WebBook (Lemmon et al., 2016). The diameter range of the sand particles was also increased accordingly.

**Fig. 4.**
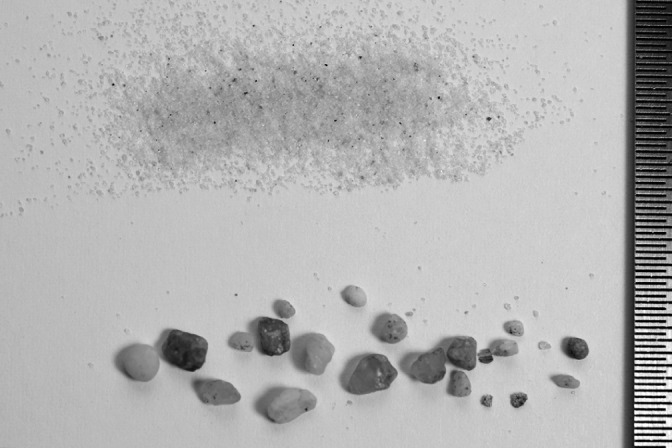
**Desert sand particles (0.1–0.5 mm diameter) and sand particles used for the experiment (0.9–4 mm diameter).**

The two parts of the model were assembled with screws, and a pump (89 ml, Sanicare, Vienna, Austria) was connected to the model with a silicone tube (Rotilabo, Karlsruhe, Germany). The model was then buried in sand (particle diameter: 0.9–4 mm, Sera, Heinsberg, Germany). A tube connected to the inert gas regulator (connected to the helium cylinder) was placed close to the model. In order to achieve a closed system, a transparent box was placed over the sand. When the box was full of helium, inhalation and exhalation were simulated manually with the help of the pump. We were unable to replicate the velocities of the ventilation patterns manually with perfect accuracy. For the inhalation, we let go as slowly as possible to fill the pump, while for the exhalation we pressed as hard as possible to blow the helium out of the pump. The inhalation velocity was higher than the calculated velocity (inhalation: *v*_exp_≈10*v*_sim_). The exhalation velocity was approximately 20% lower than that calculated from the tidal volume and the ventilation patterns (exhalation: *v*_exp_≈0.81*v*_sim_). The respiration cycle was repeated 20 times, and then the helium supply was turned off and the box was removed. Very carefully, the model was dug up by hand without tilting it. It was cleaned on the outside with a brush. Subsequently, the respiratory tract was searched thoroughly for sand particles. Visual inspection was possible because of the size of the particles and the transparent printing material (Figs 3, 4). In total, the experiment was repeated 10 times.

## RESULTS

### No sand particles in the sandfish lungs

Five sandfish specimens (two of them were preserved animals) were dissected and the respiratory tract, lungs and gastrointestinal tract were examined for sand particles. Although a substantial quantity, which makes up 40–60% of the wet mass of the skink's excrement (Hartmann, 1989), was found in the gastrointestinal tract, not a single sand particle was present in the lungs or respiratory tract.

### Histology

#### Overall description of the nasal cavity of *Scincus scincus*

**Fig. 5.**
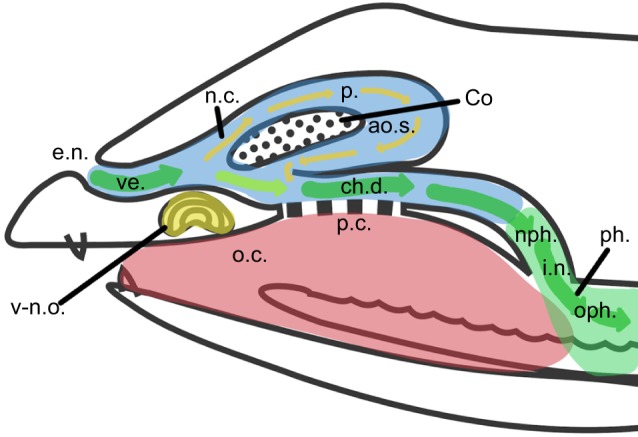
**Schematic drawing of the upper respiratory tract of the sandfish.** The blue area marks the nasal cavity (n.c.), the red area shows the oral cavity (o.c.) and the green area shows the pharynx (ph.). The airflow is represented by arrows; the green arrows show the main airflow, and the yellow ones mark the flow through the olfactory chamber. ao.s., antiorbital space; ch.d., choanal duct; Co, concha; e.n., external naris; i.n., internal naris; nph., nasopharynx; oph., oropharynx; p., proprium; p.c., palatine cleft; ve., vestibulum; v-n.o, vomero-nasal organ.

Based on literature (Parsons, 1970; Greer, 1970; Halpern, 1992) and the dissection of several specimens, the nasal cavities of *S. scincus* can be illustrated as follows (see also Fig. 5).

The upper respiratory tract constitutes two bilateral structures separated by the nasal septum on the anterior side. They merge at the pharynx (ph.). Similar to the anatomy of other lizards (Stebbins, 1948; Parsons, 1970) the nasal cavity of *S. scincus* occupies the space between the external naris (e.n.) and the internal naris (i.n.) and is composed of three main parts: the vestibulum (ve.), the proprium (p.) and the choanal duct (ch.d.). The vestibulum is a rounded channel posterior to the external naris, covered with a squamous flat epithelium and surrounded by a cavernous, possibly erectile tissue (Stebbins, 1948). At the posterior end of the vestibulum, the nasal cavity widens in cross-section and splits into the latero-dorsally lying proprium and a medio-ventrally lying choanal duct. The proprium is also dorsomedially divided by the concha (Co) into the medial space (where inhaled air enters first) and the antiorbital space (ao.s., where inhaled air enters second). Here it reconnects with the choanal duct. Most of the inhaled air, however, bypasses the proprium and flows directly through the choanal duct towards the lungs. On its ventro-medial side, the choanal duct forms a connection with the oral cavity – the palatine fissure or palatine cleft (Stebbins, 1948). Oral and nasal cavities are separated by a soft, secondary palate, supported by the pterygoid bone. Two choanae connect to the oral cavity where air reaches the trachea.

#### Description of histology

**Fig. 6.**
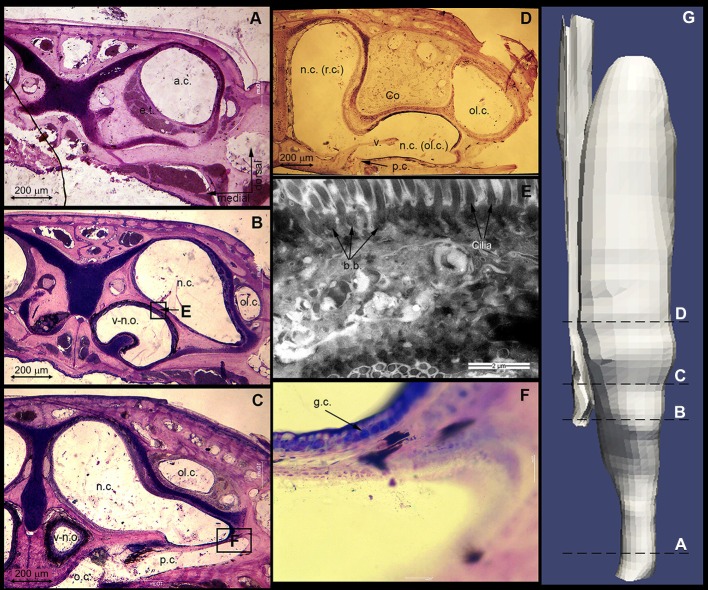
**An overview of the most characteristic histological sections and their position in the respiratory tract.** (A–D) Four characteristic histological sections: A shows the erectile tissue (e.t.), B the extension of the nasal cavity (n.c.) and its characteristic shape, C the palatine cleft (p.c.) and D the cartilage-free ‘valve’ (v.), which separates the olfactory (ol.c.) and respiratory (r.c.) chambers. (E) TEM image to visualize cilia and their basal bodies. (F) Photomicrograph at higher magnification to verify the goblet cells (g.c.). (G) Final 3D model created from the histological analysis. a.c., anterior chamber; b.b., basal bodies; Co, concha; o.c., oral cavity; v-n.o., vomero-nasal organ.

The upper respiratory tract of the sandfish (from the nostrils to the trachea) is approximately 11 mm long and approximately 3.5 mm wide at its widest point (see Fig. 1). The nasal opening has an approximate diameter of 0.84 mm. The nasal tract is narrowest (0.73 mm) at the outlet to the trachea; at its widest point we calculated a diameter of 1.4 mm (see Fig. 2, section 7d). It follows that all sand particles (0.1–0.5 mm) could easily enter the lungs through the respiratory tract. All dimensions of the respiratory tract can be found in Table S1. Thanks to the semi-thin sectioning procedure, we could process the individual sections and digitise them with a microscope camera. The images were subsequently merged to build a 3D model of one part of the bilateral respiratory tract (Fig. 6G). A selection of four characteristic segments is shown in Fig. 6A–D. The anterior chamber (a.c.) is circular, lined with stratified keratinized epithelium, and surrounded by cavernous, blood-filled tissue, i.e. erectile tissue (e.t.). Except the smooth muscles surrounding arteries, no muscle tissue was observed in this region. The nasal cavity (n.c.) expands in the lateral inferior direction towards the concha with constantly increasing cross-section. On its surface, goblet cells (g.c.) for mucus production and cilia, which can be identified by the basal bodies (b.b.) that lie on their basal side, are visible (Fig. 5E,F). This type of epithelium (nasal respiratory epithelium) begins after the vestibulum and continues in the posterior direction towards the nasopharyngeal duct. At this point, the chamber is separated into two parts: the respiratory and olfactory chambers (r.c. and ol.c., respectively). In this region, the respiratory chamber starts to narrow again to a cross-sectional area close to that of the anterior chamber. The respiratory chamber passes beneath the concha, and the olfactory chamber makes the shape of a sickle around the upper concha. The palatine cleft (p.c.) is the connection between the nasal and the oral cavity; it begins posterior to the vomero-nasal organ (v-n.o.) and ends at the choana. It forms a pocket in the oral cavity and is separated from the nasal cavity by a membrane (Fig. 6C). At the beginning of the concha the membrane changes its shape and forms a valve (v.) (Fig. 6D), a cartilage-free structure that separates the respiratory and the olfactory chambers. It seems that the connection between the nasal and oral cavities is blocked permanently by mucous secretion. Posterior to the concha, the anteorbital space begins. As can be seen in Fig. 6A, the erectile tissue is of limited size. The anterior opening can be constricted by the erectile tissue but not fully closed.

### Ventilation patterns and tidal volume

**Fig. 7.**
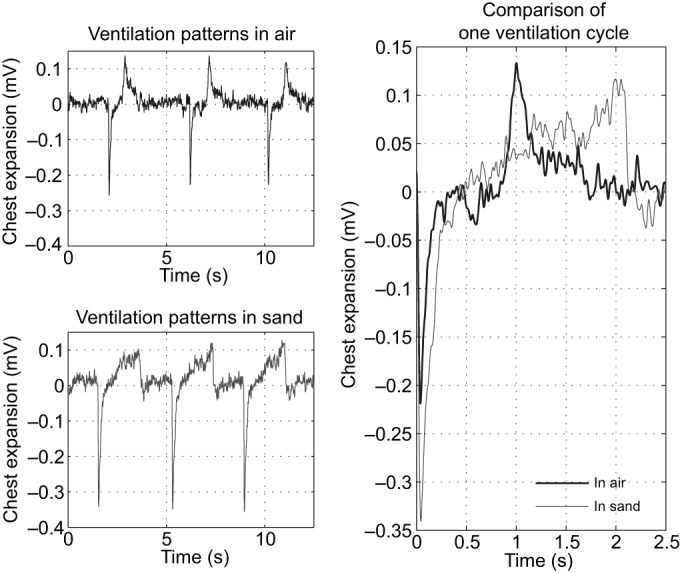
**Ventilation patterns of the sandfish below and above sand.** Exhalation is given as negative mV, whereas inhalation is given as positive mV. A comparison of the two significantly different respiration cycles shows that the first exhalation is 60% more intense below sand. The following inhalation is twice as long below sand. In total, the two cycles both last between 24 and 30 breaths min^–1^, excluding the resting period. The overall breathing frequency is approximately 15 breaths min^−1^ in both environments.

Typical results of the measurements are shown in Fig. 7. The respiration cycle is triphasic, consisting of an exhalation, an inhalation and relaxation, described as the elastic recoil of the thorax (Cragg, 1978). The cycle is completed by a resting period (Glass and Wood, 1983), also described as a breath holding (Milsom, 1984; Milsom and Vitalis, 1984). The patterns are very similar to those described by Cragg (1978), who studied the genus *Lacerta* and found a triphasic ventilation pattern and a diphasic tidal volume. Fig. 7 compares respiration cycles below and above sand. There are a number of important differences: the exhalation of the triphasic cycle is approximately 40 ms in both environments, but the intensity of the exhalation below sand is approximately 60% higher than that above sand. Above sand, exhalation lasts 38±3.8 ms; below sand it lasts 45±20 ms. In general, the exhalation can be described as cough-like. The inhalation patterns also differ dramatically: below sand, the inhalation is 2036±96 ms, above sand it is 895±106 ms. Hence, below sand, inhalation lasts more than twice as long as above sand. This significant difference is either regulated actively by the animal or caused by higher air resistance below sand because of sand particles partially blocking the nostrils or swollen erectile tissue narrowing the respiratory tract (Fig. 6A). Below sand, the elastic recoil of the thorax lasts approximately as long as the exhalation. Above sand, in contrast, the relaxation lasts approximately 800 ms and transitions slowly into the resting period. In both cases, the resting period lasts approximately 2 s and the overall breathing frequency is approximately 15 min^−1^.

**Fig. 8.**
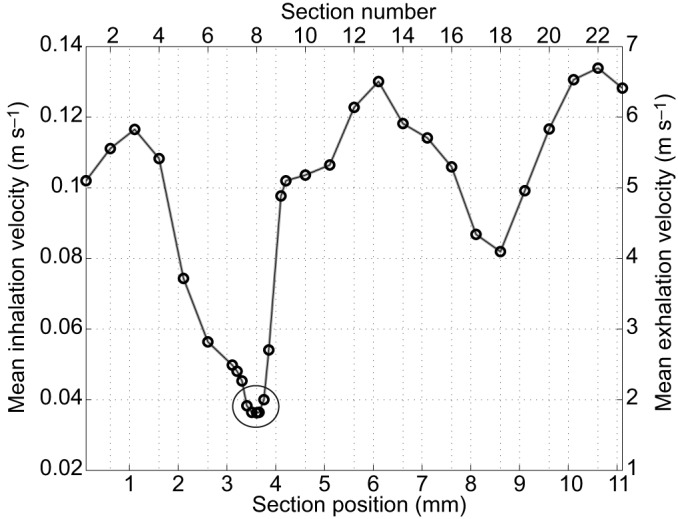
**Mean velocity fields of inhalation and exhalation for below-sand breathing in the sandfish.** The large circle indicates the area of 70% velocity drop.

The average tidal volume, determined by Vihar et al. (2015), was 227±48 μl (*n*=4); the average mass of these four specimens was 25 g. This leads to an average tidal volume (*V*_T_) of 0.908±0.192 ml 100 g^–1^, and is in good agreement with the tidal volume found by Milsom (1984), who studied the genus *Gekko*. Because the respiratory system consists of two separate tracts, the average tidal volume inhaled by one upper respiratory tract during one respiration cycle is 113.5 μl. This is a reasonable assumption, because in the family Scincidae the right and left lung sacs tend to be a mirror image (Klein et al., 2005). The mean velocities for inspiration and expiration were calculated assuming that the pressure differences detected by the piezo sensor and the inspired and expired air volumes are linearly related. Further, we assumed a linear progression of the volumetric flow (i.e. constant velocity across the initial cross-sectional area). We calculated the volumetric flow in m^3^ s^–1^. Dividing the flow by the initial cross-sectional area of the respiratory system (i.e. of the nostril), we obtained the initial velocity for inhalation and exhalation (see Fig. 8):
(7)
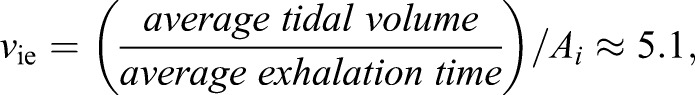

(8)
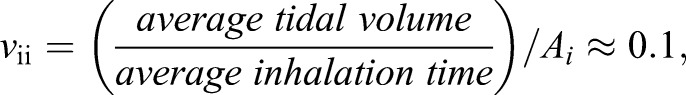

where *v*_ie_ and *v*_ii_ are the initial inhalation and exhalation velocities in m s^–1^, respectively, and *A_i_* denotes the cross-sectional area in m^2^.

### Analysis of flow dynamics

As shown in Fig. 2, the eight sections of particular interest are in the segments between sections 7 and 10. They reveal the sharp increase in the cross-sectional area of the respiratory chamber, which then goes on to decrease posterior towards the anteorbital space. As previously described, at the beginning of the concha (Fig. 2, section 8c) a cartilage-free membrane forms a valve, which separates not only the oral and nasal cavities, but also the respiratory and olfactory chambers (Fig. 2, sections 9–10) and hence leads again to a decrease in the cross-sectional area of the respiratory chamber. The results of our velocity field calculations, illustrated in Fig. 8, show that the mean velocities for inhalation are very low; more specifically, the inhalation velocities are 50 times lower than the mean exhalation velocities. The computations of the velocity field indicate a velocity drop of 70% in the sections in which mucus and cilia are present (see Fig. 8).

### Fluid mechanics experiments using the 3D-printed model

In four experiments, no sand particles were found in the respiratory tract, and one, three and four particles were found in two experiments each. In all 10 experiments, the particles were found in sectors A and B, but never posterior to sector B (Table 1, Fig. 1).

**Table 1.**
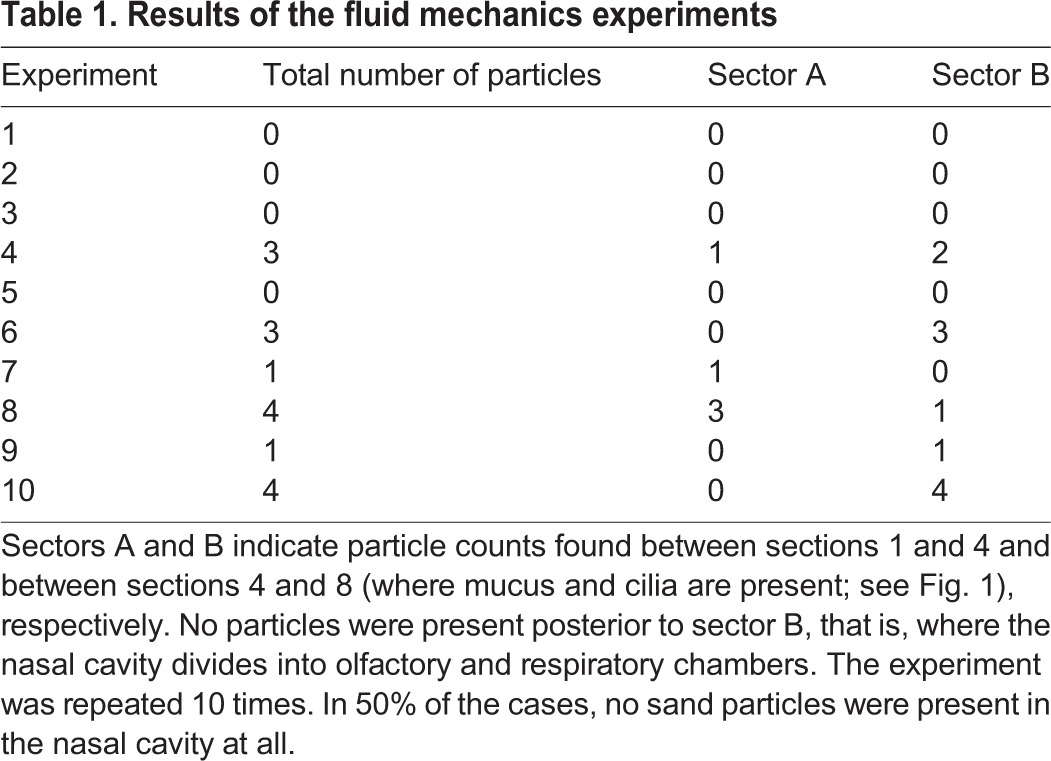
**Results of the fluid mechanics experiments**

The inhalation velocity was higher than the calculated velocity (inhalation: *v*_exp_≈10*v*_sim_). This would suggest that more particles entered the nasal cavity during the experiment because higher forces were working on the particles. The exhalation velocity was approximately 20% lower than that calculated from the tidal volume and the ventilation patterns (exhalation: *v*_exp_≈0.81*v*_sim_). The simulated exhalation was thus less intense, but, nonetheless, in 50% of the experiments no sand particles were found in the respiratory tract. We repeated the experiments with fine desert sand and found particles in all parts of the model. This suggests that the skink's respiratory tract is adapted to a certain range of sand particle size. The experiments support the computational results. If particles entered the respiratory tract, the majority was found in the section of the velocity drop (sector A, Fig. 1). No particles were present posterior to sector B, that is, where the nasal cavity divides into the olfactory and respiratory chambers (Fig. 1).

## DISCUSSION

We sought to understand the respiration and the upper respiratory system of the sandfish, *S.* *scincus*, a species that can respire without sand particles entering its respiratory organs when buried under thick layers of sand; a systematic approach helped to reveal the clues to this complex phenomenon.

In the nasal cavity we could find no filtering system, such as fine hair or narrowing, that would hinder sand particles from entering the lungs. Stebbins (1948) made similar observations when describing the upper respiratory tract of the genus *Eumeces* from the family Scincidae, the closest relative of the sandfish. He described the erectile tissue as being of limited size, and the nasal organ as poorly adapted to preventing the entry of fine particles. However, its morphology is somewhat peculiar and suggests that a dynamic filtering system exists. In order to understand the respiration dynamics, we thus measured the ventilation patterns. They show the typical triphasic cycle, as has been described before (Cragg, 1978). The patterns measured below sand differ drastically from those above sand (see Fig. 7). Interpreting the ventilation patterns below sand, the slow inhalation indicates that this is either regulated actively by the animal or caused by higher air resistance below sand because of sand particles partially blocking the nostrils or swollen tissue narrowing the respiratory tract; and the intense, cough-like exhalation suggests that the particles entering the respiratory system during inhalation are coughed out.

To calculate the velocity field, the determination of the tidal volume was necessary. This has already been measured in a previous study (Vihar et al., 2015). The obtained value is in good agreement with that found by Milsom (1984). The results of our velocity field calculations, illustrated in Fig. 8, show that the mean velocities for inhalation are very low; more specifically, the inhalation velocities are 50 times lower than the mean exhalation velocities. The computations of the velocity field indicate a velocity drop of 70% in the sections where mucus and cilia are present (see Fig. 8). Based on this finding, we formulated the following hypothesis. When particles enter the respiratory tract during inhalation (2 s), they stay in the area superior to the vomero-nasal organ owing to a great drop in velocity, where they are ‘caught’ by the mucus. The cilia transport the particles either towards the naris or towards the palatine cleft. Because of the rapid cough-like exhalation (40 ms), some of the particles are coughed out through the naris. The rest may be transported into the oral chamber through the palatine cleft and swallowed. The high amount of sand in the animal's excrement (40–60%) supports this hypothesis.

Stebbins’ (1943) studies of the lizard *Uma* led to similar conclusions. He dissected several specimens and found sand particles in the upper portion of the U-shaped nasal tube; only in a few cases did he find a small deposit of particles in the lower portion, which he assumed would be enveloped by mucus and swallowed eventually. There is a substantial difference though between the nasal organ of *Uma* and that of *S. scincus*. While *Uma* has a U-shaped cavity that prevents particles from entering the lungs, *S. scincus* has no such physiological filtering system, and mucus appears in the area of greatest cross-section – which does not seem very convincing in the first place. The huge velocity drop in this section and the results of the fluid mechanics experiment provide undeniable evidence that the sandfish has an aerodynamic filtering system.

Integrating biological studies, mathematical computations and fluid mechanics experiments, we were able to demonstrate that the sandfish has an aerodynamic filtering system to prevent sand particles from entering the lungs. We further showed that the morphology of the upper respiratory tract in combination with characteristic ventilation patterns is designed to aerodynamically filter particles of a certain size. We exclude the possibility that the sandfish cleans its nasal cavity by producing large amounts of mucus, as it lives in a highly arid region where mucus secretion would lead to an unacceptably high loss of body liquid. Future work will focus on the numerical simulation of the flow dynamics including particle simulations. Additionally, the surface structure properties of mucus and cilia will be examined in detail in order to include them in the calculations. Studying other species living in comparable areas would also be valuable, as they must deal with similar problems. It would therefore be of great interest to determine whether their respiratory systems have a similar morphology, and whether the principle of aerodynamic filtering can be applied.
